# Interpretation of anomalously long crosslinks in ribosome crosslinking reveals the ribosome interaction in stationary phase *E. coli*†

**DOI:** 10.1039/d2cb00101b

**Published:** 2022-05-16

**Authors:** Santosh A. Misal, Bingqing Zhao, James P. Reilly

**Affiliations:** Department of Chemistry, Indiana University 800 East Kirkwood Avenue Bloomington IN 47405 USA santosh.misal@nih.gov

## Abstract

Crosslinking mass spectrometry (XL-MS) of bacterial ribosomes revealed the dynamic intra- and intermolecular interactions within the ribosome structure. It has been also extended to capture the interactions of ribosome binding proteins during translation. Generally, XL-MS often identified the crosslinks within a cross-linkable distance (<40 Å) using amine-reactive crosslinkers. The crosslinks larger than cross-linkable distance (>40 Å) are always difficult to interpret and remain unnoticed. Here, we focused on stationary phase bacterial ribosome crosslinking that yields ultra-long crosslinks in an *E. coli* cell lysate. We explain these ultra-long crosslinks with the combination of sucrose density gradient centrifugation, chemical crosslinking, high-resolution mass spectrometry, and electron microscopy analysis. Multiple ultra-long crosslinks were observed in *E. coli* ribosomes for example ribosomal protein L19 (K63, K94) crosslinks with L21 (K71, K81) at two locations that are about 100 Å apart. Structural mapping of such ultra-long crosslinks in 70S ribosomes suggested that these crosslinks are not potentially formed within one 70S particle and could be a result of dimer and trimer formation as evidenced by negative staining electron microscopy. Ribosome dimerization captured by chemical crosslinking reaction could be an indication of ribosome–ribosome interactions in the stationary phase.

## Abstract 2

Ultra-long crosslinks in *E. coli* ribosomes were identified from the dimers, trimers, and tetramers using four peptide fragmentation methods. Sucrose density gradient and electron microscopy analysis confirmed ribosome dimers and trimers.

## Graphical abstract

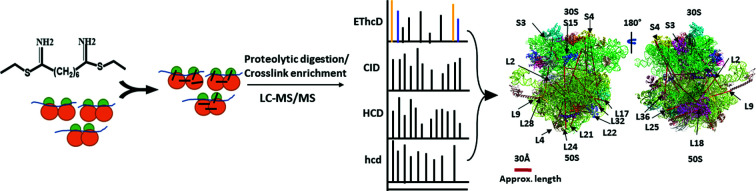

## Introduction

The bacterial protein translational machinery is primarily driven by 70S ribosome and ribosomal binding factors. Several other non-ribosomal proteins transiently bind/interact with 70S ribosomes during the process of initiation, elongation, and termination of protein synthesis.^1,2^ Both bacterial and mammalian cells control the protein synthesis *via* dimerization of 70S ribosome to a 100S ribosome.^3^ The 70S ribosome dimerization is mainly facilitated by the binding of hibernation promoting factor (HPF) and preserves the essential protein functions of the ribosome.^4^ This ribosome dimerization mainly occurs in the stationary phase of cell growth where the protein translation rate is lower due to the resting 100S ribosomes. The Cryo-EM structure of 100S ribosome dimer is determined in *Thermus thermophilus, B. subtilis*, and *S. aureus* which is facilitated by HPF and may be required for maintaining the active ribosomes for the next cycles of translation.^4–6^ This is also an important step in the cell cycle for the survival of *E. coli* cells and to avoid ribosome stalling due to amino acid scarcity. Ribosome dimers were found to be connected through the 30S subunit and the ribosomal proteins S2, S3 and S5 were critical for the 100S dimerization process.^7^ At the stationary phase of the *E. coli* growth cycle, the ribosome modulation factor (RMF) and the hibernation promoting factor (HPF) are known to reversibly bind 70S ribosomes to form and promote the formation of 100S ribosome dimerization.^7,8^ The 100S ribosome particle is translationally inactive due to blockage of peptidyl transferase center and peptide exit tunnel by binding of RMF and HPF.^9^ This inactivation state of the ribosome at the stationary phase of the bacterial cell cycle is also known as ribosomal hibernation. The RMF and HPF are released from 100S and 70S ribosome activity is restored when the cell regrowth is initiated.^10,11^ The existence of ribosome dimerization was also detected during the exponential phase of the cell cycle in *Staphylococcus aureus* and *Lactobacillus paracasei*.^7,12^ Ribosome dimerization evidence and mechanism are well studied in *E. coli* and *Thermus thermophilus*.^7,13^ All reported evidence of the ribosome dimerization is based on the presence of 100S particles in a sucrose density gradient and the cryo-transmission electron microscopy (cryo-TEM).^9,11^ Recently, the ribosomal proteins S2 and S3 were discovered as the point of contact between two 70S ribosome particles in 100S dimerization.^4^ However, this does not provide direct evidence of interacting amino acid residues. To gain more insight into the involvement of ribosomal proteins and respective residues we designed the study to capture the ribosome dimer and tried to rationalize the ultra-long crosslinks by combining the sucrose density gradient, electron microscopy, chemical crosslinking, and high-resolution mass spectrometry.

The XL-MS techniques have been actively utilized in the field of protein biochemistry and proteomics to study the protein–protein interaction and their topologies.^14,15^ The usage of distance constraints obtained from the XL-MS in structural biology is rapidly increasing to gain insights into low-resolution protein structures.^16,17^ Most of the chemical crosslinkers include the *N*-hydroxysuccinimide (NHS) esters as the main reactive group that can selectively react with primary amine groups of the proteins.^18^ These are commonly used crosslinkers that covalently link the interacting functional group within the approximate distance in the protein. In the present study, we have used an ETD-cleavable homo-bifunctional thioimidate cross-linking reagent, diethylsuberthioimidate (DEST), which can effectively crosslink the proteins under physiological conditions.^19–22^ DEST has an 11 Å spacer arm and crosslinks to primary amines without altering their native basicity. The total cross-linkable distance is ∼24 Å including lysine side chains (∼6.5 Å) is similar to commercially available crosslinkers. Recently, many MS cleavable and enrichable crosslinkers were introduced for definitive identifications of cross-links and to reduce the challenges in the conventional peptide fragmentation methods.^18,23–25^ Modern mass spectrometers with Electron Transfer Dissociation fragmentation with supplementary activation of HCD (EThcD) led to the best sequence coverage for highly charged cross-linked peptides.^26^ These advancements in crosslinking mass spectrometry are utilized in this study to obtain more insight into ribosome dimerization and involvement of ribosomal proteins and respective amino acid residues at the stationary phase of the bacterial cell cycle. In this study, we present evidence of ribosome dimer formation at the stationary phase of *E. coli* that potentially explains the formation of ultra-long crosslinks observed in the ribosome crosslinking.

## Results and discussion

### Chemical crosslinking of the ribosome captured the dimers and trimers

**Fig. 1:**
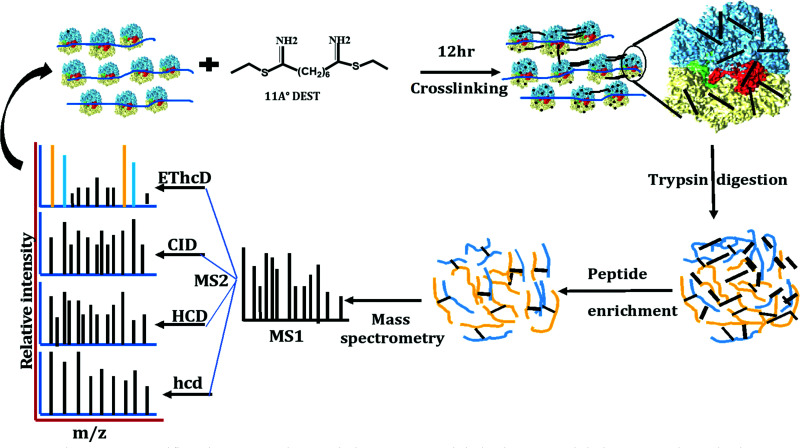
Ribosome XL-MS workflow. The stationary phase *E. coli* ribosomes are crosslinked with DEST. Crosslinked proteins are digested with trypsin followed by crosslinked peptide enrichment using SCX chromatography and high-resolution mass spectrometry data acquisition with four different methods of precursor fragmentation.

**Fig. 2:**
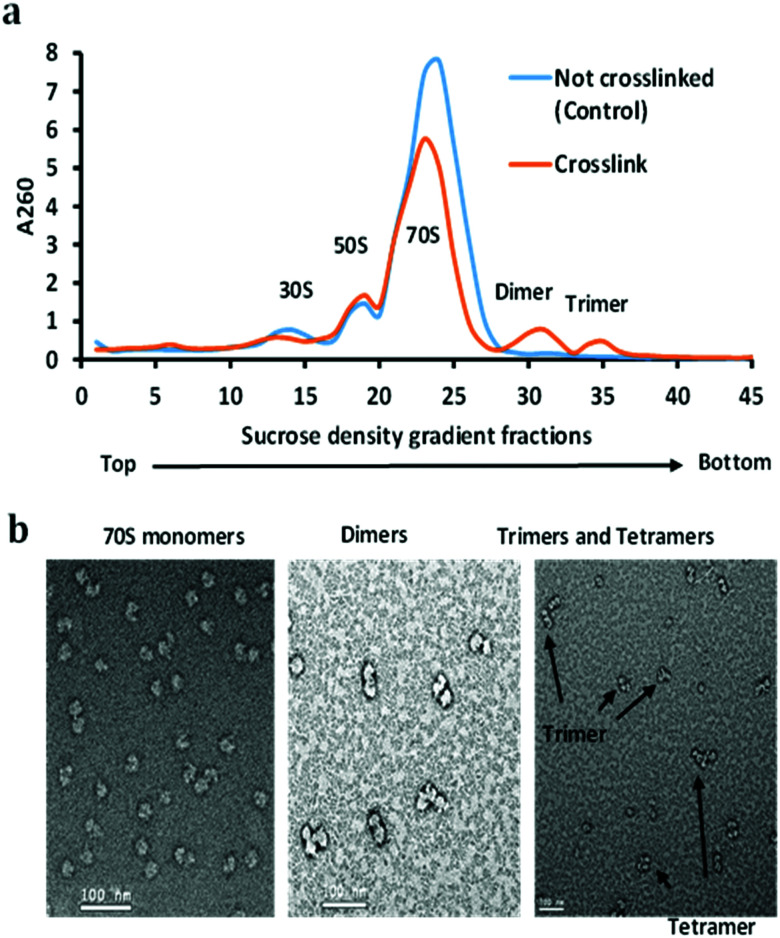
(a) Sucrose density gradient fractionation of non-crosslinked (control) and the crosslinked ribosome. Both crosslinked and non-crosslinked ribosomes were purified with a 10–50% sucrose gradient. Dimer and trimer peaks were the result of crosslinking. (b) 70S monomeric ribosome particles, dimer, and trimer peaks were analyzed using negative staining electron microscopy.

The stationary phase *E. coli* K12 cells were lysed, diluted, and divided into two parts, one part was used as a control (not crosslinked), and the other was used for the crosslinking reaction with DEST. The ribosomes were isolated and purified by the sucrose cushion and sucrose density gradient centrifugation methods from crosslinked and control cell lysate. The sucrose cushion centrifugation was employed to collect all ribosomes followed by a 10–50% sucrose density gradient. The sucrose density gradient profile peaks corresponding to the 70S ribosome monomer, dimer, and trimer were isolated and analysed by negative staining electron microscopy and high-resolution mass spectrometry. The overview of the ribosome crosslinking and mass spectrometry analysis workflow is given in Fig. 1. The control sucrose density gradient profile which has no crosslinker added only yields three peaks corresponding to 30S, 50S, and 70S ribosome particles (Fig. 2(a)). Interestingly, we did not observe the polysome peaks in the control ribosome sucrose density gradient. Polysomes are the multiple 70S ribosome particles held together by mRNA. The stationary phase of the *E. coli* sucrose density gradient generally comprises more polysomes than the 70S monomer when the cell lysate was directly layered on a sucrose density gradient.^27^ The absence of polysome may be due to the two-step ribosome isolation and purification procedure. In contrast, the sucrose density gradient of the crosslinked ribosomes yields two additional peaks at the place of the polysomes that were pooled separately and analysed by negative staining cryo-electron microscopy and mass spectrometry (Fig. 2(b)). These peaks appeared to be dimers, trimers, and tetramers generated after the crosslinking reaction with DEST. Previously, it was shown that the non-translating 70S ribosome particles form 100S dimers with HPF and RMF at the stationary phase and under stressed conditions.^27^ In this experiment, we did not observe any crosslinks of HPF, or RMF to ribosomal proteins; however, we did observe a few peptides and dead-ends of HPF and RMF in the crosslinked sample which suggests the transient interactions of these proteins to the stationary phase ribosome (Table S1, ESI†). The 100S particle may have dissociated during the two-step ribosome isolation but the interacting ribosomes which were held by mRNA were captured by DEST crosslinking. The presence of crosslinked 70S particles was confirmed by negative staining cryo-electron microscopy. The additional evidence of the 30S and 50S link *via* ribosomal proteins S19 and L31 was obtained by high-resolution mass spectrometry analysis of these crosslinked proteins. The deep analysis of dimer and trimer peaks yields some ultra-long crosslinks that are consistent with electron microscopy observations. The data strongly suggest that upon chemical crosslinking, transiently interacting ribosomes are captured in the form of dimers, trimers, and tetramers at the stationary phase of *E. coli.*

### DEST crosslinked ribosome dimers do not dissociate in a low magnesium sucrose density gradient

**Fig. 3:**
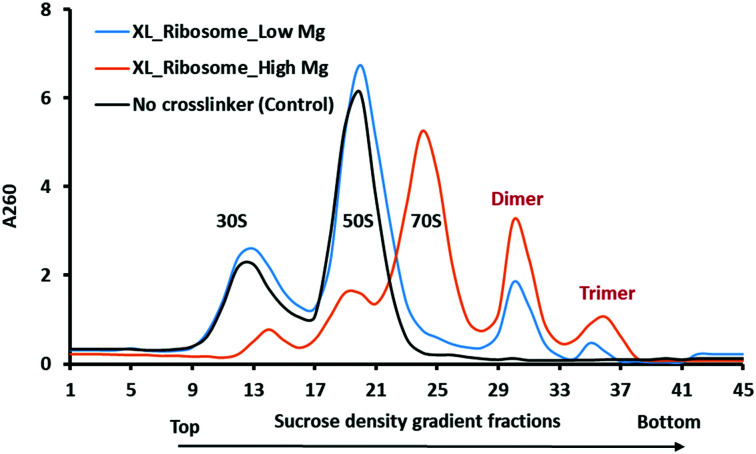
Sucrose density gradient of crosslinked and non-crosslinked (control) ribosomes with high (10.5 mM, orange curve) and low magnesium ions (1 mM). Crosslinked ribosomes (XL) do not completely dissociate in a low magnesium sucrose gradient (blue curve). In the control sample, 70S ribosomes dissociate into 30S and 50S subunit particles in low magnesium as shown by the black curve.

The stationary phase of the bacterial cell is the equilibrium between the numbers of dividing and dying cells when the protein translation rate is much lower than that of the exponential phase. The majority of ribosomes are resting but still held on mRNAs as polysomes.^28^ We did not observe the polysomes peaks in the sucrose density gradient using our conditions of ribosome isolation. However, upon DEST crosslinking of the cell lysate yield dimers and trimers peaks in a sucrose density gradient. Furthermore, we checked whether the resultant dimers and trimers dissociate in a low magnesium sucrose density gradient before analysing them *via* electron microscopy and mass spectrometry. The divalent magnesium ions (Mg^2+^) are essential for neutralizing the charge on ribosomal RNA (rRNA) and stabilization of 70S ribosome particles. The lower concentration or complete removal of Mg^2+^ causes ribosomes to disassemble into the 30S and 50S particles.^29^ We applied the dimer and trimer samples on the low magnesium sucrose gradient to see how the highly crosslinked dimer and trimer particles dissociate into 70S monomers or 30S and 50S particles. Interestingly, the crosslinked ribosome, dimer, and trimer partially dissociate into the 30S and 50S particles in the low magnesium sucrose density gradient (Fig. 3). The partial dissociation of the crosslinked ribosome, dimer, and trimer particles confirmed that the non-crosslinked particles dissociated but the highly crosslinked ribosome particles did not completely dissociate. It also suggests that a greater number of inter-molecular crosslinked proteins keep ribosome particles intact in low magnesium. The crosslinked ribosomes isolated by a high magnesium sucrose density gradient were further analysed *via* high-resolution mass spectrometry with previously developed four fragmentation methods.^22^ The mass spectrometry analysis of cross-linked peptides from ribosome dimer and trimer fractions identifies some ultra-long crosslinks of ribosomal proteins, that are far beyond the cross-linkable distance of DEST.

### Chemical crosslinking could capture the ribosome–ribosome interactions

Mass spectrometry data acquisition of crosslinked peptides using the four-fragmentation mass spectrometry method and in-house developed crosslinked peptide search algorithm were employed for confidant identification. This novel approach used to obtain the complete fragmentation information of the crosslinked peptide by CID, HCD, hcd, and ETD is extensively discussed previously.^22,30^ The target and decoy 70S ribosomal protein database was created from the most recent *E. coli* K12 proteome downloaded from the Uniprot database. The 70S ribosome crystal structure (4YBB) coordinates from the PDB database were added to the searching algorithm to accurately calculate the distance of cross-linked residues. The distance restraints obtained from XL-MS analysis are particularly important to determine the molecular proximity, topology, and relative orientation of individual ribosomal proteins in the 70S ribosome.^31^ XL-MS provides definitive binary interaction data (*e.g.*, subunit A is close in space to subunit B) and spatial restraints between proteins with a resolution of several amino acids at the primary sequence level (limited by the location of cross-linkable residues). These restraints are in the range of 7–30 Å, with a median distance of approximately 15 Å for the most commonly used lysine-reactive reagents and slightly shorter for carboxyl-reactive hydrazides and zero-length crosslinks.^24^ We observed several cross-linkable (short distance) intra- and inter-molecular crosslinks from the dimer and trimer ribosomes most of which were previously observed (Fig. S1 and Table S2, ESI†).^20,32^

Lauber and Reilly isolated the ribosomes from *E. coli* K12 and cross-linked them with DEST. They observed 52 intra-protein and 19 interprotein crosslinks within the 30S or 50S subunits. However, the linkage between these two subunits proteins was not observed.^20^ Ji *et al.* reported 132 intra-protein and 84 interprotein crosslinks confidently identified with 1% FDR from the ribosome using the XLSearch algorithm.^32^ The majority of these short crosslinks also observed and identified confidently in the current experiment. Multiple interprotein crosslink of L9–L28 at different amino acid residues observed by Lauber and Reilly and Ji *et al.* also observed in the current experiment. In the current experiment, we observed more intra-protein crosslinks than interprotein crosslinks which is consistent with the previously reported crosslinks. However, there are few short intra- and interprotein crosslinks that Lauber and Reilly observed were not observed in the current experiment or *vice versa* due to the different experimental conditions. Tuting *et al.* reported 115 intra-protein cross-links and 71 interprotein cross-links that are within the cross-linkable distance threshold (30 Å for intra- and 37.5 Å for inter-molecular cross-links).^33^

**Fig. 4:**
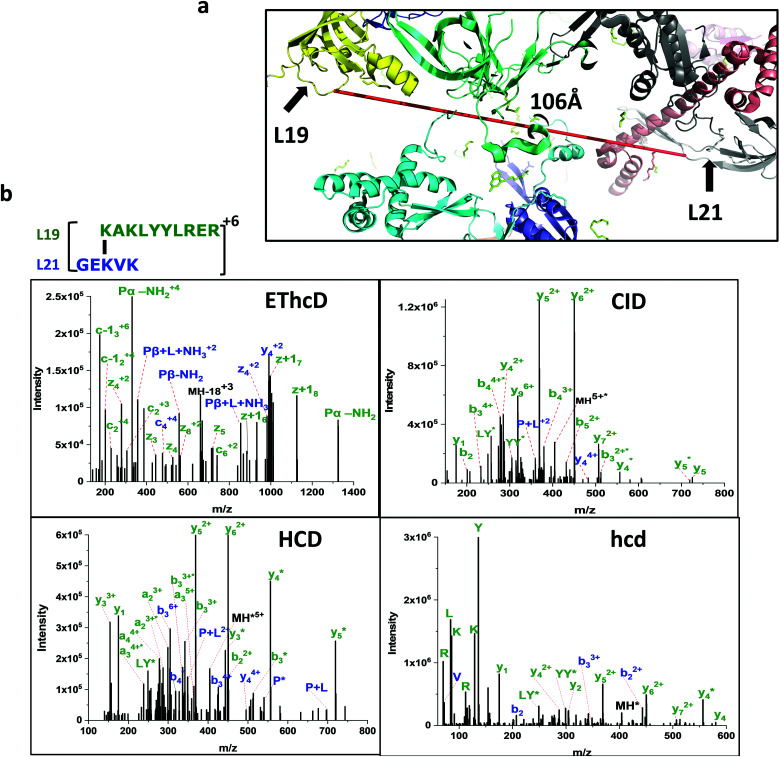
(a) Ultra-long crosslink between ribosomal proteins L19 and L21. (b) The crosslinked peptide is confidently identified by four different fragmentation methods. CID and HCD spectra give a series of b and y ions. The EThcD spectrum confirms the partially fragmented crosslinked peptide mass pairs and additional confirmation by the presence of immonium ions in the hcd spectrum.

**Fig. 5:**
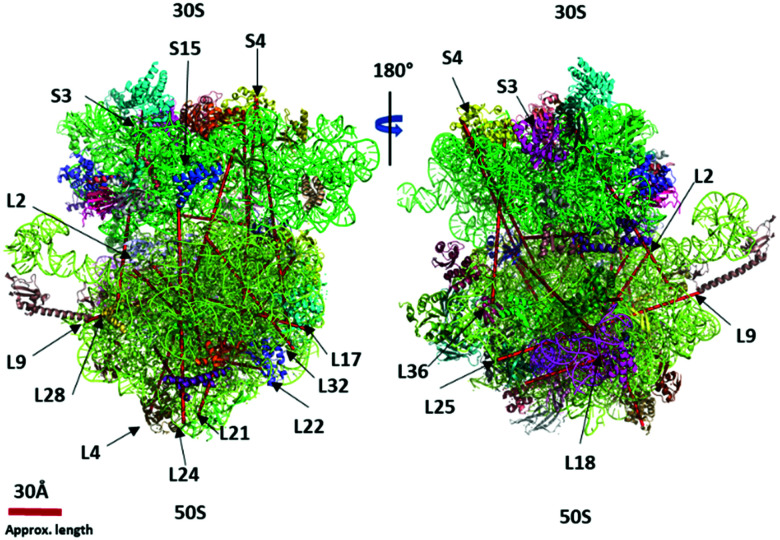
The ultra-long crosslinks (>40 Å) are mapped in the 70S ribosome crystal structure (PDB 4YBB) using the PyXlinkViewer plugin in PyMOL v2.4.1 – Schrodinger, LLC.

In our current approach to confidently identify the crosslinks, we fragmented the precursor ions with CID, HCD with different energies and mass ranges, and ETD with supplemental HCD energy (EThcD). One precursor yielded four MS2 spectra that were searched for crosslinked peptide pairs. DEST crosslinked peptides preferentially dissociate at the amidine group in ETD yielding mass pairs of Peptide-NH2 (P-NH2) and Peptide + Linker + NH3 (P + L + NH3) of both constituent peptides that were the prerequisite to qualify for the real DEST crosslinks. CID and HCD MS2 spectra provide a series of fragment ion information that is needed to identify the crosslinked peptide. The hcd MS2 with a lower mass range and higher energy yields more internal fragment ions and immonium ions which provides the complementary information of the individual residues present in the crosslinked peptides. This four-fragmentation mass spectrometry approach provides a simple but reliable and confident identification of cross-links. We reliably identified about 100 short-distance intra- and intermolecular crosslinks in 70S ribosome dimers and trimers. The distances (Cα–Cα) within the range of 5–30 Å of cross-linked residues were considered the short crosslinks and mapped separately in the 70S ribosome crystal structure (Fig. 4 and 5). The crosslinking distance of the unresolved proteins or residues in the crystal structure are not calculated and labeled as NA in the final crosslink list. For instance, the initial 10 residues of ribosomal protein S11 were not resolved in the 4YBB crystal structure. We observed the crosslink between the residues K3–K14 and A2–K14 of S11. It was previously shown that the initiator methionine is cleaved by aminopeptidase and the second residue alanine was methylated.^34^ Our observation of initiator methionine removal and crosslinked second residue alanine with K14 shows the flexibility of the N-terminal domain of S11. This could be the reason that the S11 N-terminal domain was not resolved in the crystal structure. Additionally, the crosslink distance between L1 and L15 was not calculated in Table S2 (ESI†) due to the absence of L1 in the 4YBB crystal structure.

We observed 17 interprotein ultra-long crosslinks in dimer and trimer ribosomes. The linkage between the 30S and 50S subunits found between ribosomal protein S19 and L31 situated on the surface of the interface were observed crosslinked *via* lysine (S19 (K29)–L31 (K70)) (Table 1). The lysine (K70) is situated at the C-terminal domain of L31 and is still unresolved in the crystal structure. The C-terminal domain of L31 was found to be highly flexible and have two distinct conformations.^33^ This is consistent with our observed short crosslink between K62 and K70 of ribosomal proteins L31 (Table S2, ESI†) and the crosslink between L31 and S19. The L31 C-terminal domain interacts simultaneously with S19 and itself at K62–K70. In the 4YBB 70S ribosome crystal structure the L31 is not resolved, and therefore the distance between S19 and L31 is not calculated. This linkage has recently been mapped by data-driven homology modeling and the distance has been calculated.^33^ Our short (L31, K62–K70) and long crosslinks ((S19, K29–L31, K70)) corroborate with their observation and confirm the binding of the L31 C-terminal domain to the S19 during the assembly of the ribosome.

**Table 1:** Ultra-long crosslinks (≥40 Å) observed in ribosomal proteins

Trap	*m*/*z*	*z*	Peptide 1	Peptide 2	X-link length (Å)	Protein 1	Protein 2
2	504.497	5	[M*1]K[136.10]VR(A)	(R)L[K83]GNTGENLLALLEGR(L)	243.1	L36	S4
2	497.898	5	(K)[K16]ILKQAKGYYGAR(S)	(M)[E2]TIAKHR(H)	41.5	L20	L22
2	663.633	4	(R)E[K9]SVEELNTELLNLLR(E)	[M1]PKIK(T)	82.1	L29	L35
3	465.275	3	(−)M*[153.13]AH[K4]K(A)	(K)AA[K555]GE(−)	NA	L27	S1
4	457.503	4	(R)[K63]ISNGEGVER(V)	(R)[K81]HYR(K)	99	L19	L21
4	435.76	4	(R)[K17]LQELGATR(L)	(R)[K10]EQGK(G)	59.8	L18	L25
4	438.765	4	(R)K[153.13]*VIA*[K57](−)	(R)SE[K121]AEAAAE(−)	NA	L32	L17
4	546.619	3	(K)[K48]DHHSR(R)	(R)FEDG[K91]K(V)	271	S15	L24
4	971.91	3	(K)QSRV[K156]AALELAEQR(E)	(R)HEII[K35]TTLPK(A)	320.9	S4	L17
4	518.703	5	(K)VEK[136.10]AVESGDK[K29]PLR(T)	(R)FNIPGS[K70](−)	NA	S19	L31
4	912.732	4	(R)SHDALTAVTSLSVDK[155.09]TSGE[K37]HLR(H)	(K)C[K7]PTSPGR(R)	100	L32	L2
4	533.634	3	(R)SE[K121]AEAAAE(−)	(M)[a2]KGIR(E)	NA	L17	L33
4,9	648.128	4	(K)VEKAVESGD[K28]K[136.10]PLR(T)	(R)FNIPGS[K70](−)	NA	S19	L31
5	377.741	4	(R)K[136.10]GR[K233](−)	(R)VSA[K54]GMR(V)	NA	S3	L28
6,9	559.342	4	(R)V[K156]AALELAEQR(E)	(R)ADRIL[K98]R(T)	318.6	S4	L22
8	420.863	5	(R)GEKVKIV[K76]FR(R)	(K)TRSN[K261]R(T)	99	L21	L2
9	438.274	4	(R)[K94]AKLYYLR(E)	(R)GE[K71]VK(I)	106	L19	L21

### Ultra-long intermolecular crosslinks

About 100 short-distance crosslinks were observed in the ribosomal proteins within the cross-linkable distance (5–30 Å) of the DEST crosslinker (Table S2, ESI†). The DEST crosslinker has an 11 Å spacer arm which leads to ∼24 Å cross-linkable distance. The crosslinks beyond the cross-linkable distance are often rationalized by the flexibility of the protein domains in the solution and live cells. The observed ultra-long crosslinks cannot be explained by the flexibility of protein domains, but rather suggest the novel ribosome–ribosome interactions at the stationary phase of the *E. coli*. The crosslink between 30S subunit ribosomal protein S4 (K156) and 50S subunit ribosomal protein L17 (K35) is 171 Å long. Both proteins are located on the opposite side of the ribosome and this linkage is not possible within one ribosome particle. In order to crosslink with these residues, the other ribosome particle must be in its proximity which strongly suggests that there could be transient interaction between two ribosome particles. At the same time, S4 (K156) also crosslinks with the ribosomal protein L22 (K98) which is also approximately 177 Å. Ribosomal proteins L17 and L22 are closer and may interact with S4 simultaneously. The ribosomal particles orient in such a way that S4 can easily crosslink with L17. Recently, S4 protein was demonstrated to be more flexible than other ribosomal proteins and forms a capped ring around the RNA-exit tunnel with Nus-factors SuhB.^35^

Lauber *et al.* demonstrated the 30S subunit ribosomal protein S1 crosslink with small subunit proteins S2, S3, S6, S7, S9, S18, S19, and S21.^21^ Recently, Tuting *et al.* remodeled the S1 interactions and found new crosslinks with S10, L9, and L1 ribosomal proteins.^33^ We found the C-terminal domain of S1 (K555) crosslink with the large subunit 50S ribosomal protein L27 (K4). Since the S1 protein is not structurally characterized in many of the 70S ribosome crystal structures including 4YBB, the crosslink L27 (K4) and S1 (K555) is not mapped and not able to rationalize.

We observed multiple redundant ribosomal protein L19 (K63) crosslinks with L21 (K81) and L19 (K94) crosslinks with L21 (K71) (Fig. 4 and 5). These proteins are about 100 Å away and situated opposite to each other in the 70S ribosome crystal structure. Their linkage is not possible in one ribosome structure. Similarly, the ribosomal L17 (K121) crosslinked with L33 (A2) where the starting methionine of L33 is cleaved off, and the N-terminal of the second residue alanine is available to crosslink with ribosomal protein L17 (K121) which is also over 100 Å apart. The accurate distance was not calculated due to the lack of the first three residues of L33 in the 4YBB crystal structure. Also, the C-terminal domain (K233) of small subunit protein S3 is crosslinked with 50S subunit protein L28 (K54). Interestingly, this is another linkage observed between the small subunit and the large subunit but the C-terminal domain of L33 is not resolved in the crystal structure and is not able to map the crosslink distance.

**Fig. 6:**
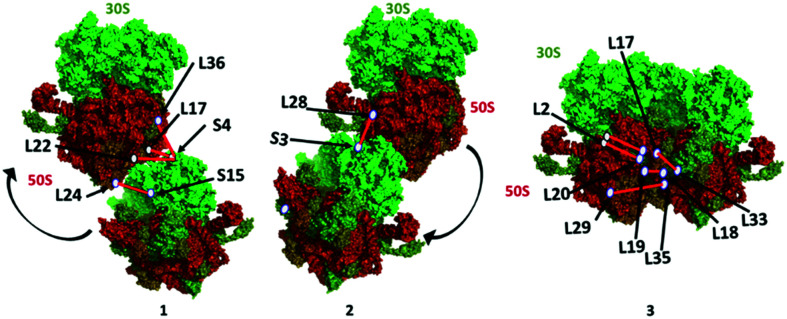
Proposed ribosome dimer interaction models (1, 2, and 3) constructed using the PDB 4YBB crystal structure in PyMOL v2.4.1 – Schrodinger, LLC. The locations of ribosomal proteins/residues and the crosslink distance are approximate based on the crystal structure.

The presence of an ultra-long crosslink in the 70S ribosome proteins strongly supports the inherent plasticity of the 70S monomer in response to a variety of stress conditions. The stationary phase is a typical nutrient limiting stage where 70S ribosomes form the 100S dimer after binding of HPF and RMF. Upon reliving of stress conditions, 100S particles dissociate into the 30S and 50S subunits.^36^ We crosslinked at the stationary phase and captured the dimers and trimers that were still resting on mRNA or accumulated in the stationary phase before completely dissociating into the 30S and 50S subunits. Therefore, this data indicates the presence of 70S ribosome particles in proximity or transiently interacting in the stationary phase (Fig. 6).

## Conclusions

Inter- and intra-molecular ribosomal protein interactions are essential in the translation process and adequately studied by chemical cross-linking mass spectrometry. However, the interactions of translating or stationary phase ribosomes are not known except for the 100S particle formation. We provide the evidence of ribosome dimerization and their interactions and systematically explain the ultra-long crosslinks generated by DEST in the stationary phase of the *E. coli* using the combination of the sucrose density gradient, electron microscopy, four fragmentation mass spectrometry methods, and an in-house developed crosslinking search algorithm. The translating and resting ribosomes on mRNA were captured by chemical crosslinking in the form of the dimer, trimer, and tetramer as evidenced in a sucrose density gradient, electron microscopy, and ultra-long crosslink analysis. The ribosome interactions need to be further investigated for their significance in the translation process.

## Experimental

### Bacterial growth and culture conditions

*E. coli* K12 MG1655 cells were grown for 12 h at 37 °C in 500 mL of LB broth. Cells were pelleted by centrifugation at 8000 rpm for 10 min using the JA10 Beckman Coulter rotor at 4 °C. The cell pellet was washed twice with 20 mM HEPES, 100 mM NH_4_Cl, pH7.4 and dissolved in lysis buffer (20 mM HEPES, 100 mM NH_4_Cl, 10.5 mM Mg acetate, 0.5 mM EDTA, 5 mM 2-mercaptoethanol, 3 mM PMSF and Roche cOmplete protease inhibitor tables 1/10 mL). The cells were lysed using Emulsifier EmulsiFlex-C3 at 10 000 PSI for 6 cycles at 4 °C. The lysate was collected and centrifuged at 15 000 rpm for 45 min in a Beckman Coulter JA-20 rotor. The protein concentration was determined by Bradford assay using BSA as a standard. The cell lysate was aliquoted and diluted with cross-linking buffer (25 mM HEPES-NH_4_OH, 100 mM NH_4_Cl, 10.5 mM Mg acetate, pH 7.4@20 °C).

### Chemical crosslinking and ribosome isolation

DEST was synthesized by following the protocol by Lauber and Reilly and stored in a vacuum desiccator at 4 °C.^19^ DEST powder was dissolved in crosslinking buffer and added to cell lysate at a 20 : 1 (DEST to protein) ratio.^19^ The reaction mixture was incubated for 6 h at room temperature with a moderate vortex. The crosslinking reaction was quenched by adding 250 mM NH_4_Cl. Excess and hydrolysed DEST was removed by Amicon Ultra 10 K centrifugal filter (Millipore, Germany). The crosslinked cell lysate was layered on a 1.1 M sucrose cushion in Spedding buffer (20 mM HEPES-KOH pH 7.5 at 4 °C, 20 mM NH_4_Cl, 10.5 mM Mg acetate, 0.5 mM EDTA, 5 mM 2-mercaptoethanol, 3 mM PMSF) and centrifuged at 36 600 rpm for 18 h at 4 °C using a 70ti Beckman Coulter rotor in Optima XPN ultracentrifuge. The ribosome pellet formed at the bottom of the centrifuge tube was washed at least twice and dissolved in Spedding buffer. Ribosome concentration was determined by measuring the absorbance at 260 nm with Thermo Scientific™ NanoDrop 2000 UV-Vis spectrophotometer. 10 units (A260) of ribosomes (approx. 500 μg) was layered on a 10–50% sucrose density gradient and centrifuged at 19 000 rpm for 18 h at 4 °C using a SW41 rotor in Optima XPN ultracentrifuge. The gradient was fractioned with a homemade fractionator and A260 was measured. The fractions corresponding to 70S monomer, dimer, and trimer were pooled separately and divided into two parts. One part of these pooled fractions was analysed by crosslinking mass spectrometry and the other was analysed by negative staining electron microscopy. A control ribosome sample was prepared similarly by the two-step centrifugation method without a crosslinker. A low magnesium sucrose density gradient was performed to dissociate ribosomal subunits similarly in Spedding buffer using 1 mM Mg acetate instead of 10.5 mM Mg acetate. The crosslinking reaction and sucrose density gradient were repeated at least 5 times with different DEST : protein ratios (20 : 1, 100 : 1, 200 : 1, 500 : 1, and 1000 : 1).

For mass spectrometry analysis, the pooled fractions were precipitated with 10% TCA at 4 °C. The precipitated proteins were centrifuged at 14.1 RCF and washed thrice with ice-cold acetone. Proteins were dried and dissolved in 100 mM ammonium bicarbonate, pH 7.8. Trypsin was added to the protein solution at a 1 : 50 ratio and incubated at 37 °C for 18 h. The cross-linked peptides were enriched by SCX and reverse phase chromatography as described earlier by Lauber and Reilly.^20^ In brief, the crosslinked peptides were injected into the SCX column (TSKgel SP-NPR, 4.6 mm × 35 mm, Tosoh Bioscience) and the eluent was captured on 10 different C18 trap columns (Hypersil-Keystone Javelin, 1.0 mm × 20 mm, ThermoFisher Scientific). The C18 trap columns were desalted by 5% ACN, 0.1% TFA in water for 10 min each at a flow rate of 0.3 mL min^−1^ and then eluted with 90% ACN, 0.1%TFA in 10 Eppendorf tubes. The eluate from each trap column was dried in a vacuum. These enriched peptide fractions of the SCX were then resuspended in 0.1% FA in water for mass spectrometry analysis. The mass spectrometry experiments were done once.

For electron microscopy analysis, the excess sucrose from the pooled fractions of the 70S, dimer, and trimer peaks was removed using Amicon 100 kD molecular weight filter in separate vials. Each sample was diluted 25× using post-crosslinking buffer (20 mM HEPES-KOH pH 7.5 at 4 °C, 20 mM NH_4_Cl, 10.5 mM Mg acetate) and mixed with freshly prepared 2% uranyl acetate in a 1 : 1 ratio. The mixture was immediately applied to fresh glow discharged continuous carbon EM grids. The solution that remained on the grid was blotted out with Whatman filter paper and allowed to air dry for 1 h. The images were recorded on a JEOL JEM 3200FS microscope.

### High-resolution mass spectrometry and database search

Enriched crosslinked peptide solution was dried in a speed vacuum evaporator and redissolved in 25 mM ammonium bicarbonate, pH 7.5, and acidified with 0.1% formic acid (FA). Approximately 1 μg of peptides were loaded on a nanoACQUITY UPLC symmetry C18 trap column (waters) in 95% solvent A (0.1% FA in water (HPLC grade)) and 5% solvent B (0.1%FA in acetonitrile (HPLC grade)). Peptides were eluted and separated using a 60 min gradient from 3 to 48% of solvent B at a flow rate of 300 nL min^−1^ on a C18 ACQUITY UPLC HSS T3 column (Waters). The eluent from the C18 column was electrosprayed in Thermo Orbitrap Lumos mass spectrometer in positive ion mode using 1.8 kV voltage. Peptides with the precursor mass in the *m*/*z* range 300 to 2000 with 3 to 8 charges were selected for further fragmentation. The resolution for MS1 was set at 120 000 FWHM and the AGC target was set to 4.0 E5. Precursor ions with intensity threshold 5.0 E6 were fragmented with three different fragmentation methods including ETD, CID, and HCD with variable fragmentation energy. Precursor ions with a charge more than 3+ were first fragmented with ETD with 50 ms reaction time supplemented with 15% HCD energy. The same precursor ion was fragmented with 35% CID energy. The HCD fragmentation was done with 35% energy with a scan range of 140–2000 *m*/*z* and 45% energy with a scan range of 68–800 *m*/*z*. The raw data were converted to the Mascot generic format (mgf) using the MSConvert program from ProteoWizard.

### XL-MS data analysis

The mgf and extracted MS1 files were submitted to the Xlink matcher program, an in-house built program that computes metrics such as precursor mass errors, numbers of peaks matched for each constituent peptide, percentages of ion intensities matched to each constituent peptide, and scores for tentative identifications from each fragmentation such as CID, HCD, and EThcD. Crosslinked peptides were searched with *E. coli* K12 ribosomal protein target database downloaded from UniProt (https://www.uniprot.org/proteomes/UP000000625) and randomized decoy database. Precursor mass tolerance was set to 5 ppm and fragment mass tolerance was set to 0.02 Da. The enzyme digestion was set as full trypsin and variable modifications were set as methionine oxidation and deamidation (NQ). The minimum peptide length was 4 and three missed cleavages were allowed. Maximum crosslink mass was set to 5000 Da. The crosslinked length (Xlink length) of the residues was calculated using the 70S ribosome crystal structure (PDB 4YBB) downloaded from the RCSB protein data bank. The *XYZ* coordinates (Cα) of all residues were downloaded in the standard mmCIF format and manually curated the ribosomal proteins. The simplified coordinate.text file was loaded on the Xlink matcher program. The identifications from EThcD were searched with a minimum of two mass pair peaks from the crosslinked peptide pair. The output text files were sorted based on the maximum intensity match, peptide mass pairs, and maximum peaks matched. Crosslinked peptides were manually checked for consistent identification from four fragmentation methods. The cross-linked peptides with no mass pairs in EThcD and no consistent identification with four different fragmentation methods were removed from the final list. The crosslinked residues were mapped and visualized in the 70S ribosome crystal structure (PDB 4YBB) using the PyXlinkViever plugin tool in PyMOL.^37^

## Author contributions

SAM and JPR designed the study. BZ synthesized the DEST crosslinker and SAM performed the experiments. SAM, BZ and JPR analysed the data. SAM, JPR wrote the manuscript and all authors contributed to its reviewing and editing.

## Conflicts of interest

There are no conflicts to declare.

## Supplementary Material

CB-003-D2CB00101B-s001
