# Supplementary material for: Interpretation of anomalously long crosslinks in ribosome crosslinking reveals the ribosome interaction in stationary phase E. coli
Source: RSC Chem Biol. 2022 May 16;3(7):886–94. doi: 10.1039/d2cb00101b (PMC9257603; doi:10.1039/d2cb00101b)
Supplement: CB-003-D2CB00101B-s001 [file CB-003-D2CB00101B-s001.pdf]

Electronic Supplementary Material (ESI) for RSC Chemical Biology.
This journal is © The Royal Society of Chemistry 2022

Supporting information

Interpretation of Anomalously Long Crosslinks in Ribosome Crosslinking reveals the ribosome interaction in stationary phase E. coli

Santosh A. Misal*; Bingqing Zhao; James P. Reilly

Department of Chemistry, Indiana University, Bloomington, IN 47405

Correspondence- Santosh A. Misal, santosh.misal@nih.gov

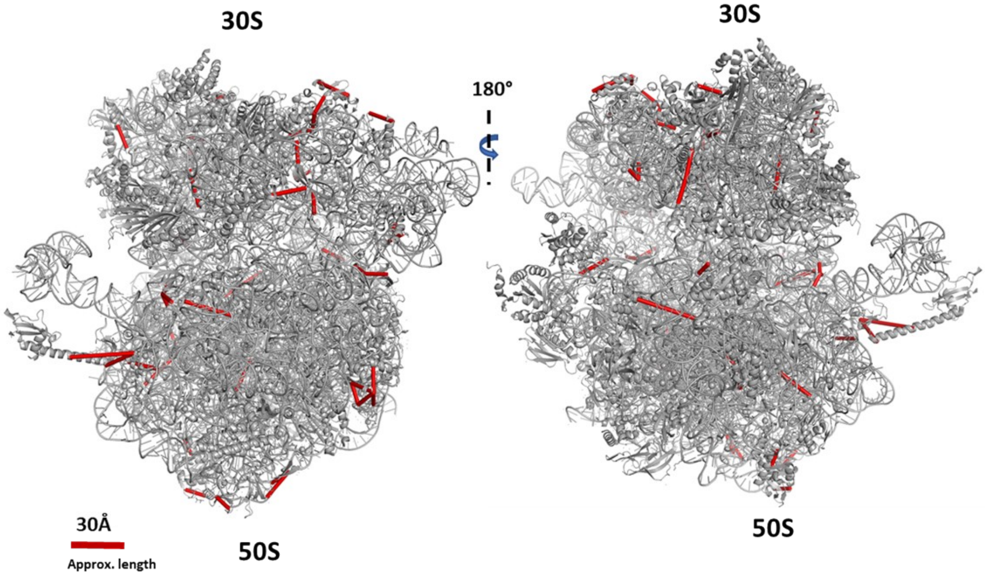

Figure S1: The short crosslinks (<40Å) are mapped in the 70S ribosome crystal structure (PDB 4YBB) using the PyXlinkViewer plugin in PyMOL v2.4.1 - Schrodinger, LLC. The complete list is given in Table S1.

Table S1. Peptides and dead-ends of HPF and RMF were observed in the analysis.

Trap	Fragmentation method	Peptide	Dead-end mass	Protein
2	CID	(K)FA[K26]LEQYFDR(I)	153.1216	P0AFX0|HPF_Ribosome hibernation promoting factor
3	hcd	(K)HKDKLKQH(-)		P0AFX0|HPF_ Ribosome hibernation promoting factor
3	HCD	(R)S[K26]EM*CPYQTLNQR(S)	155.0946	P0AFW2|RMF_Ribosome modulation factor
3	hcd	(R)S[K26]EM*CPYQTLNQR(S)	155.0946	P0AFW2|RMF_Ribosome modulation factor
4	ETD	(R)LERAHQR(G)		P0AFW2|RMF_Ribosome modulation factor
4	ETD	(R)LERAHQR(G)		P0AFW2|RMF_Ribosome modulation factor
4	CID	(R)EFVTAKFAK(L)		P0AFX0|HPF_Ribosome hibernation promoting factor
4	hcd	(R)EFVTAKFAK(L)		P0AFX0|HPF_Ribosome hibernation promoting factor
4	hcd	(K)DKL[K93]QH(-)	136.0992	P0AFX0|HPF_Ribosome hibernation promoting factor
4	CID	(M)[Q2]LNITGNNVEITEALREFVTAKFAK(L)	258.169	P0AFX0|HPF_Ribosome hibernation promoting factor
6	hcd	(R)QLT[K87]HKDK(L)	136.0971	P0AFX0|HPF_Ribosome hibernation promoting factor
6	ETD	(K)H[K89]DKLKQH(-)	136.0987	P0AFX0|HPF_Ribosome hibernation promoting factor
6, 7	ETD, CID, HCD	(R)QLT[K87]HK(D)	155.0954	P0AFX0|HPF_Ribosome hibernation promoting factor
9	hcd	(R)QLT[K87]HKDK(L)	136.0973	P0AFX0|HPF_Ribosome hibernation promoting factor

Table S2. The short crosslinks (<40Å) were observed in the dimer and trimer ribosomes.

Trap Number	m/z	Peptide 1	Peptide 2	Protein 1	Protein 2	XL length (Å)
Trap1						
	350.80	4(R)AIQSE[K16]AR(K)	4(R)[K19]HNASR(R)	S20	S20	5.2
	546.29	4(K)SNSETI[K104](-)	4(R)FEDG[K91]K(V)	L24	L24	18
	471.03	4(R)V[K156]AALELAEQR(E)	4(K)[K151]QSR(V)	S4	S4	8.07
	478.78	4(R)SGSI[K74]SPIWR(S)	4(R)Q[K63]GTGR(A)	L4	L4	19.75
	431.77	4(R)[K11]ILPDPK(F)	4(R)[K100]AGFVTR(D)	S7	S9	14.1
	605.34	4(R)VFQTHSPVVDSISV[K87]R(R)	4(R)[K63]ISNGEGVER(V)	L19	L19	13.22
	632.74	4(R)VGFFNPIASE[K46]EEGTRLDLDR(I)	4(K)EVN[K80]AA(-)	S16	S16	17.4
Trap2						
	428.04	4(R)HIGGGH[K59]QAYR(I)	4(R)IVDF[K68]R(N)	L2	L2	26.9
	340.23	4(R)[K76]FGLSR(I)	4(R)I[K83]VR(E)	S14	S14	12.71
	471.03	4(R)V[K156]AALELAEQR(E)	4(K)[K151]QSR(V)	S4	S4	8
	487.95	4(R)FNIPGS[K70](-)	4(R)FN[K62]R(F)	L31	L31	NA
	474.26	4(R)[K183]VEADCR(A)	4(R)TD[K265]FIVR(R)	L2	L2	8.2
	464.08	4(R)RLP[K63]FGFTSR(K)	4(R)[K70]AAITAEIR(L)	L15	L15	20.8
	593.07	4(K)[K42]NIEFFEAR(R)	4(R)FWVESE[K44]R(F)	L9	L28	22.8
	591.35	4(R)L[K83]GNTGENLLALLEGR(L)	4(R)[K185]PER(S)	S4	S4	14.59
	673.41	4(R)GAL[K106]SILSELVR(Q)	4(K)[K99]MYR(G)	L4	L4	10.6
	745.01	(R)E[K167]PTWLEVDAGKMEGTFK[136.10[R(K)	(R)V[K156]AALELAEQR(E)	S4	S4	16.4
	570.32	4(R)L[K83]GNTGENLLALLEGR(L)	4(R)NYY[K77]EAAR(L)	S4	S4	11.8
	642.86	4(R)ALLAAFDFPFR[K179](-)	4(R)GLSA[K120]SFDGR(G)	L5	L5	15.9
	985.87	4(R)AELEA[K57]LAEVLAAANAR(A)	4(R)FWVESE[K44]R(F)	L9	L28	33.13
	591.34	(R)L[K83]GNTGENLLALLEGR(L)	(R)[K185]PER(S)	S4	S4	14.59
	1037.91	(R)RAELEA[K57]LAEVLAAANAR(A)	(R)FWVESE[K44]R(F)	L9	L28	33.13
	348.88	(R)LGAKGI[K150]VEVSGR(L)	(M)[&2]HIEK(Q)	S3	S5	39.6
	605.05	4(K)LLDYL[K71]R(K)	4(R)VVE[K81]AVL(-)	S15	S17	18
	410.00	(-)MAKLT[K6]R(M)	(R)GH[K36]GQK(S)	L1	L15	NA

778.68	3(R)RAELEA[K57]LAEVLAAANAR(A)	3(R)FWVESE[K44]R(F)	L9	L28	33.138
639.93	3(R)SDLSADINEHLIVELYS[K206](-)	3(R)NYY[K77]EAAR(L)	S4	S4	13.52
546.29	4(K)SNSETI[K104](-)	4(R)FEDG[K91]K(V)	L24	L24	18
593.07	4(K)[K42]NIEFFEAR(R)	4(R)FWVESE[K44]R(F)	L9	L28	22.78
623.15	4(R)RAELEA[K57]LAEVLAAANAR(A)	4(R)FWVESE[K44]R(F)	L9	L28	33.138
380.23	4(R)TD[K265]FIVR(R)	4(R)SN[K261]R(T)	L2	L2	7.3
318.20	4(R)QG[K62]IWIR(V)	4(R)T[K8]FR(K)	L16	L16	32.2
474.26	4(R)[K183]VEADCR(A)	4(R)TD[K265]FIVR(R)	L2	L2	8.2

Trap4

618.37	3(R)[K108]PELDAK[153.13[[LVADSITSQLER(R)	3(R)LGA[K147]GIK(V)	S3	S3	14.614
567.74	4(R)RDDEVIVLTG[K17]DK(G)	4(K)VIVEGINLV[K43]K(H)	L24	L24	15.1
519.05	4(K)[K62]GIDTVLAELR(A)	4(R)ARGE[K77]Y(-)	L28	L28	19.228
482.31	3(R)LIVVE[K123]FSVEAPK(T)	3(K)LLAQ[K137]LK(D)	L4	L4	13.163
593.07	4(K)[K42]NIEFFEAR(R)	4(R)FWVESE[K44]R(F)	L9	L28	22.78
902.95	4(R)TT[K43]LHVHDENNECGIGDVVEIR(E)	4(R)VVSD[K16]MEK(S)	S17	S17	14.8
772.91	4(K)AAAQ[K49]AFNEMQPIVDR(Q)	4(K)[K34]VYAAIEAGDK(A)	S20	S20	9.855
594.82	4(R)E[K167]PTWLEVDAGK(M)	4(K)MEGTF[K183]R(K)	S4	S4	13.532
705.89	2(R)NIPVGSTVHNVEM[K147]PGK(G)	2(R)TD[K265]FIVR(R)	L2	L2	14.6
598.81	4(R)E[K167]PTWLEVDAGK(M)	4(K)M*EGTF[K183]R(K)	S4	S4	13.532
776.91	4(K)AAAQ[K49]AFNEM*QPIVDR(Q)	4(K)[K34]VYAAIEAGDK(A)	S20	S20	9.855
485.26	4(R)GALDCSGV[K108]DR(K)	4(K)YGV[K120]R(P)	S12	S12	10
756.42	4(R)VFQTHSPVVDSISV[K87]R(R)	4(R)[K63]ISNGEGVER(V)	L19	L19	13.228
379.61	4(R)[K183]VEADCR(A)	4(R)TD[K265]FIVR(R)	L2	L2	8.2
605.34	4(R)VFQTHSPVVDSISV[K87]R(R)	4(R)[K63]ISNGEGVER(V)	L19	L19	13.228
474.26	4(R)[K183]VEADCR(A)	4(R)TD[K265]FIVR(R)	L2	L2	8.2
488.31	4(K)VIVEGINLV[K43]K(H)	4(K)DKG[K21]R(G)	L24	L24	18.8
589.08	4(R)DHTLFA[K62]ADGK(V)	4(R)[K17]LQELGATR(L)	L27	L18	15.512
507.83	4(R)K[155.09[[VIA[K57](-)	4(R)HEII[K35]TTLPK(A)	L32	L17	13.203
542.32	3(R)G[K24]VK[136.10[[NVLSSGK(V)	3(K)SNSETI[K104](-)	L24	L24	20
455.24	4(R)GALDCSGV[K108]DR(K)	4(R)[K111]QAR(S)	S12	S12	9.3
555.81	4(R)HEII[K35]TTLPK(A)	4(R)SE[K121]AEAAAE(-)	L17	L17	9.2

481.07	3(R)AK[136.10[[ASAV[K54]R(H)	3(R)EFYE[K40]PTTER(K)	S21	S21	21.312
585.34	4(R)K[155.09[[VIA[K57](-)	4(R)SE[K121]AEAAAE(-)	L32	L17	14.5
471.03	4(R)V[K156]AALELAEQR(E)	4(K)[K151]QSR(V)	S4	S4	8.078
546.29	4(K)SNSETI[K104](-)	4(R)FEDG[K91]K(V)	L24	L24	18.4
541.34	4(R)[K108]PELDAK(L)	4(R)LGA[K147]GIK(V)	S3	S3	14.614
585.01	2(R)K[154.11[[VIA[K57](-)	2(R)SE[K121]AEAAAE(-)	L32	L17	14.5
546.29	4(K)SNSETI[K104](-)	4(R)FEDG[K91]K(V)	L24	L24	18.4
438.76	4(R)K[153.13[[VIA[K57](-)	4(R)SE[K121]AEAAAE(-)	L32	L17	14.5
533.64	4(R)SE[K121]AEAAAE(-)	4(R)[K53]VIAK(-)	L17	L32	14.5
409.97	4(K)SNSETI[K104](-)	4(R)FEDG[K91]K(V)	L24	L24	18.4
457.51	4(R)[K63]ISNGEGVER(V)	4(R)TG[K106]AAR(I)	L19	L19	14.464
438.25	4(R)AIQSE[K16]AR(K)	4(R)[K19]HNASR(R)	S20	S20	5.308
985.88	4(R)AELEA[K57]LAEVLAAANAR(A)	4(R)FWVESE[K44]R(F)	L9	L28	33.138
919.15	2(R)DNEIVA[K78]LFNELGPR(F)	2(R)SE[K121]AEAAAE(-)	L17	L17	26.4
874.50	3(K)AIISDVNASDEDRWNAVL[K47]LQTLPR(D)	3(K)KGPFIDLHLL[K17]K(V)	S14	S19	13.6
1037.91	3(R)RAELEA[K57]LAEVLAAANAR(A)	3(R)FWVESE[K44]R(F)	L9	L28	33.138
799.66	3(R)SDLSADINEHLIVELYS[K206](-)	3(R)NYY[K77]EAAR(L)	S4	S4	13.52
603.32	3(R)TRDNEIVA[K78]LFNELGPR(F)	3(R)SE[K121]AEAAAE(-)	L17	L17	26.4
608.17	2(K)AIISDVNASDEDRWNAVL[K47]LQTLPR(D)	2(K)YFA[K23]R(A)	S14	S14	13.847
712.64	3(R)L[K83]GNTGENLLALLEGR(L)	3(R)NYY[K77]EAAR(L)	S4	S4	11.851
Trap5					
350.80	4(R)AIQSE[K16]AR(K)	4(R)[K19]HNASR(R)	S20	S20	5.308
406.26	4(R)[K108]PELDAK(L)	4(R)LGA[K147]GIK(V)	S3	S3	14.614
474.26	4(R)[K183]VEADCR(A)	4(R)TD[K265]FIVR(R)	L2	L2	8.2
788.13	4(R)L[K83]GNTGENLLALLEGR(L)	4(R)[K185]PER(S)	S4	S4	14.592
778.68	4(R)RAELEA[K57]LAEVLAAANAR(A)	4(R)FWVESE[K44]R(F)	L9	L28	33.138
409.97	4(K)SNSETI[K104](-)	4(R)FEDG[K91]K(V)	L24	L24	18.4
686.39	3(R)[K14]QVSDGVAHIHASFNNTIVTITDR(Q)	3(M)[a2]KAPIR(A)	S11	S11	NA
722.56	3(R)TT[K43]LHVHDENNECGIGDVVEIR(E)	3(R)VVSD[K16]MEK(S)	S17	S17	14.8
591.35	3(R)L[K83]GNTGENLLALLEGR(L)	3(R)[K185]PER(S)	S4	S4	14.592
799.66	3(R)SDLSADINEHLIVELYS[K206](-)	3(R)NYY[K77]EAAR(L)	S4	S4	13.52

	533.64	4(R)SE[K121]AEAAAE(-)	4(R)[K53]VIAK(-)	L17	L32	14
	438.76	4(R)K[153.13[[VIA[K57](-)	4(R)SE[K121]AEAAAE(-)	L32	L17	14.5
	546.29	4(K)SNSETI[K104](-)	4(R)FEDG[K91]K(V)	L24	L24	18.4
Trap6						
	686.39	3(R)[K14]QVSDGVAHIHASFNNTIVTITDR(Q)	3(M)a[K3]APIR(A)	S11	S11	NA
	623.15	3(R)RAELEA[K57]LAEVLAAANAR(A)	3(R)FWVESE[K44]R(F)	L9	L28	33.138
	406.07	3(R)K[153.13[[VIA[K57](-)	3(R)HEII[K35]TTLPK(A)	L32	L17	13.203
	474.26	3(R)[K183]VEADCR(A)	3(R)TD[K265]FIVR(R)	L2	L2	8.2
	447.48	3(K)A[K150]K[153.13[[QSR(V)	3(R)V[K156]AALELAEQR(E)	S4	S4	8.678
	438.25	4(R)AIQSE[K16]AR(K)	4(R)[K19]HNASR(R)	S20	S20	5.308
	463.03	4(K)EAK[136.10[[K[K177](-)	4(R)RPEPY[K158]GK(G)	L6	L6	18.6
	474.26	4(R)[K183]VEADCR(A)	4(R)TD[K265]FIVR(R)	L2	L2	8.2
	619.12	4(R)EKAK[136.10[[[K151]QSR(V)	4(R)V[K156]AALELAEQR(E)	S4	S4	8.078
	554.84	4(K)AK[136.10[[[K151]QSR(V)	4(R)V[K156]AALELAEQR(E)	S4	S4	8.078
Trap7						
	451.53	4(R)FV[K30]HPIYGK(F)	4(R)IY[K87]R(K)	S17	S8	12.2
	340.23	4(R)[K76]FGLSR(I)	4(R)I[K83]VR(E)	S14	S14	12.718

NA- not available, The 2,3,4 numbers in peptides 1 and 2 columns indicate the EThcD crosslinked peptide mass pairs.
